# Reproduction in porcine circovirus type 2 (PCV2) seropositive gilts inseminated with PCV2b spiked semen

**DOI:** 10.1186/1751-0147-54-51

**Published:** 2012-08-31

**Authors:** Giuseppe Sarli, Federico Morandi, Serena Panarese, Barbara Bacci, Domenico Ferrara, Carlo Bianco, Laura Fusaro, Maria Laura Bacci, Giovanna Galeati, Michele Dottori, Paolo Bonilauri, Davide Lelli, Giorgio Leotti, Thais Vila, Francois Joisel, Gordon Allan, Cinzia Benazzi, Fabio Ostanello

**Affiliations:** 1 Department of Veterinary Medical Science, University of Bologna, Via Tolara di Sopra 50, 40064, Ozzano Emilia Bologna, Italy; 2 Istituto Zooprofilattico Sperimentale della Lombardia e dell’Emilia-Romagna (IZSLER) – Section of Reggio Emilia, Via Pitagora 2, 42100, Reggio Emilia, Italy; 3 Istituto Zooprofilattico Sperimentale della Lombardia e dell’Emilia Romagna (IZSLER) –Section of Brescia, Via A. Bianchi 9, 25124, Brescia, Italy; 4 Merial Italia Spa, Strada 6, Palazzo E/5, 20090 Milanofiori, Assago, Milan, Italy; 5 Merial SAS, 29 av Tony Garnier, 69007, Lyon, France; 6 Queens University, Belfast, UK

**Keywords:** Swine, Porcine circovirus type 2, Semen, Infection

## Abstract

**Background:**

Since 1999, field evidence of transplacental infection by porcine circovirus type 2 (PCV2) and reproductive failure has been reported in pigs. The objective of this study was to evaluate the clinical and pathological consequences of PCV2 infection in conventional PCV2-seropositive gilts by insemination with PCV2b-spiked semen.

**Results:**

Six PCV2 seropositive gilts were inseminated with PCV2b-supplemented semen (infected) and three animals with semen and cell culture medium (controls). Only three out of the six infected animals were pregnant by ultrasonography on day 29 after insemination, while two out of the three controls were pregnant. One control gilt aborted on day 23 after insemination but not due to PVC2. Viraemia was demonstrated in four out of six infected and in one control gilt that became infected with PCV2a. Anti-PCV2 antibody titres showed dynamic variations in the infected group throughout the study. Among infected gilts, the animal with the lowest anti-PCV2 titre (1/100) at the beginning of the experiment and another that reached a similar low value during the experiment showed evident seroconversion over time and had also PCV2 positive foetuses. One placenta displayed mild focal necrosis of the chorionic epithelium positively stained by immunohistochemistry for PCV2 antigen.

**Conclusions:**

PCV2-seropositive gilts can be infected with PCV2 after intrauterine exposure and low maternal antibody titre may increase the probability of a foetal infection.

## Background

Porcine circovirus type 2 (PCV2)-related conditions cause economic losses to the pig industry around the world. Apart from post-weaning multisystemic wasting syndrome (PMWS), PCV2 is associated with a number of conditions collectively known as porcine circovirus diseases (PCVD) [1-6].

Recently, a new line of studies have focused on PCV2-associated reproductive failure, including irregular return to oestrus, failure of pregnancy progressing to abortion, or reduced litter size with PCV2 being detected in aborted and stillborn foetuses [7-9]. Although the main route of PCV2 transmission is thought to be faecal-oral [10], some studies have speculated on the role of artificial insemination, even though this route has never been proved [11,12]. Transplacental spread of PCV2 has been demonstrated [13,14] and the vertical transmission of PCV2 to conceptuses is found to be due to a prolonged viraemia in pregnant sows with low anti-PCV2 antibody titre [15,16]. Materno-foetal transmission can occur via free viral PCV2 particles or cell-mediated viraemia [13]. Prolonged duration of PCV2 viraemia in the sow seems to increase the probability of vertical transmission [14,15].

The role of viraemia was also implicated by Mateusen and others [16] who proved that exposure to PCV2 during the earliest stages of embryonic development with an intact zona pellucida (ZP) did not cause infection. On the contrary, blastocyst exposure after the ZP phase led to immunolabelling of PCV2 in placenta, mesonephros and neural groove of viable and non-viable embryos, the latter showing a stronger positivity [16]. The virus can also be isolated not only in semen but also in oocytes of infected sows [17-20] and can be also transmitted to the conceptus as a consequence of genital tract affection [19].

To date, several studies have investigated the role of PCV2 in experimental infection of pregnant swine such as surgical trans-uterine viral inoculation [21,22], oro-nasal infection of specific pathogen-free (SPF) [18] or seronegative conventional sows [14], insemination with PCV2-spiked semen of PCV2 seronegative or SPF sows [15,23,24], whereas investigations employing the intrauterine route with infected semen in conventional PCV2 antibody-positive sows are lacking. The available literature mainly describes foetal lesions in PCVD reproductive pathology and the distribution of PCV2 in the genital tract, oocytes and embryos, but uterine or placental lesions are poorly or not documented. In addition, Madson and others [15] suggest persistent infection could increase the probability of vertical transmission to foetuses, reporting that experimentally infected PCV2 seronegative sows gave birth to polymerase chain reaction (PCR)-positive live-born piglets with detectable serum anti-PCV2 antibodies in the presuckling state, attributable to in utero PCV2 transmission. Therefore, the objective of this study was to evaluate infection of unvaccinated PCV2-seropositive gilts with artificially PCV2-infected semen. The interferences exerted by PCV2 from days 0 to 55 of pregnancy were also evaluated.

## Methods

### Statement of animal care

The experimental procedures were approved by the Animal Experimentation Ethical and Scientific Committee - *Alma Mater Studiorum* of the University of Bologna and subsequently submitted to and approved by the Italian Ministry of Health. The study was carried out in accordance with European legislation regarding the protection of animals used for experimental and other scientific purposes (Council Directive 86/609/EEC).

### Animals

Nine healthy prepubertal, conventional, six-month-old, Large White gilts (96.4 ± 4.3 kg body weight) were purchased from a local PCV2-infected breeding farm and allowed to acclimatise for five days; a Large White boar was obtained from the Veterinary Faculty stable facility. The gilts had been vaccinated with an inactivated gE-deleted vaccine against Aujeszky’s Disease virus (ADV) and Porcine Parvovirus (PPV) as instructed by the leaflet. The animals were numbered by ear tags and randomly divided into two groups: an infected group (six animals: I1, I2, I3, I4, I5, I6) and a control group (three animals: C1, C2, C3). The groups were housed in separate rooms where each gilt was kept in an individual 5.5 m^2^ box equipped with an individual water nipple and feeding trough. Gilts were fed twice a day 2 kg/day/animal of a balanced ration especially formulated for gestation. During feeding, rectal temperature and clinical appearance was recorded. All personnel entering the rooms used disposable protective clothing.

### Virus

The virus used was a PCV2b strain isolated from an outbreak of PMWS in Italy and genotyped as reported by Hesse and others [25]. It was propagated in circovirus-free PK15 cells and its identity was confirmed by PCR analysis and reactivity with specific PCV2 monoclonal antibodies. The viral suspension titre was determined by growing serial dilutions in circovirus-free PK15 cell monolayers, followed by immunofluorescent labelling for viral antigen.

### Semen processing

A sperm-rich fraction of ejaculate was collected by gloved hand technique from a Large White mature boar, evaluated for motility, morphology and concentration by microscopy, and extended in equal volumes of Androhep^TM^ (Minitub, Tiefenbach, Germany). The semen was tested by real-time PCR (RT-PCR) to rule out any PCV2 DNA [25]. To evaluate a potential negative effect of viral suspension on sperm quality, viability and motility were analyzed before and after mixing with viral suspension. Viability was assessed with a live/dead sperm viability kit (Molecular Probes, Inc., Eugene, OR, USA) and at least 200 spermatozoa per sample were scored with a Nikon Eclipse E 600 epifluorescence microscope (Nikon Europe BV, Badhoeverdop, The Netherlands). The percentage of overall motile spermatozoa was subjectively evaluated using a pre-warmed glass slide under a contrast-phase microscope at 400× magnification. Nine standard doses (3 × 10^9^ spermatozoa/100 ml) were prepared in Androhep^TM^; six doses containing 10 ml of viral suspension (10^3.9^ TCID_50_/ml PCV2; total viral dose: 10^4.9^ TCID_50_) in minimal essential medium (MEM) and three doses 10 ml of MEM (control, sham dose); the infectious dose of PCV2b used was similar to doses used elsewhere [15]. Doses were then incubated for one hour at room temperature before artificial insemination (AI).

### Experimental design

The day of artificial insemination (DAI) was set to day 0. The animals were treated with 1500 IU/gilt of equine chorionic gonadotropin (eCG, Folligon®, Intervet, the Netherlands) on DAI −4 and 60 h later (i.e. DAI −2) with 750 IU hCG (Corulon®, Intervet, the Netherlands), both intramuscularly (IM), to induce oestrus synchronization and superovulation. Forty hours later (i.e. on DAI 0), the gilts manifested glaring standing reflex to man, and were artificially inseminated once with a standard semen dose [26] utilizing sterile Melrose catheters: six gilts were inseminated using semen mixed with the viral suspension (as above) and three control gilts using semen supplemented with 10 ml of MEM to obtain sham control semen doses. Approximately at DAI +20-21 and subsequent days, animals were examined to evaluate oestrus signs. At DAI +29, ultrasound was performed to determine if the gilts were pregnant. Empty subjects (C2, I5, I3 and I6) underwent pharmacological euthanasia after anaesthesia, whereas the pregnant animals were euthanized on DAI +52 (C3), +55 (I1 and I2) +56 (C1 and I4). The pharmacological protocol adopted for euthanasia was: 1) 8 ml of Stresnil® (Janssen Animal Health, Beerse, Belgium) IM; 2) 25 ml of Ketavet100® (Intervet Productions S.r.l., Milan, Italy) IM after 20 min.; 3) 10 ml of 50 mg/ml pentothal sodium® (Intervet Productions S.r.l., Milan, Italy) intravenously (IV) after 20 min. 4) and 10 ml Tanax® (Intervet Italia, Latina, Italy) IV.

### *In vivo* samplings

Vaginal, nasal and rectal swabs, and blood samples were collected from all gilts prior to PCV2 infection, on DAI −2 and then weekly thereafter until DAI +52. The serum obtained was stored at −20°C until assessments. Blood samples were not collected at DAI +14 so as not interfere with the maternal recognition of pregnancy (13–18 days).

### Progesterone serum evaluation

Serum progesterone (P4) was analysed by validated radioimmunoassay as previously described [27] to determine pregnancy before ultrasonography on DAI +28. Briefly, aliquots of 200 μl were extracted with 5 ml petroleum ether. After centrifugation, ether was collected and dried under a N2 stream. Dried ether extracts were resuspended in 1 ml phosphate buffer and aliquots of 100 μl were then assayed. The sensitivity of the assay was 1.0 pg/tube. The intra- and inter-assay coefficients of variation were 6.3 and 8.6%, respectively. The results are expressed as ng/ml.

### Serology

Serum antibody titres to PCV2, PPV, ADV and porcine respiratory and reproductive syndrome virus (PRRSV) were determined by testing serial dilutions of each serum. Antibodies to PCV2 were detected by an in-house competitive ELISA based on monoclonal antibodies against PCV2 [28]. Tenfold dilutions of the sera from 1/10 to 1/10000 were tested and the highest dilution giving at least a 75% inhibition of the control reaction was considered PCV2 positive. PPV antibodies were determined by testing fourfold dilutions (from 1/4 to 1/256) of each serum with an in-house competitive ELISA performed using the neutralizing monoclonal antibody (MAb) 3C9D11H11 from ATCC (ATCC number CRL 1745). The serological analyses to ADV were performed by two MAb-based in-house competitive ELISAs for anti-gB and anti-gE antibody detection [29]. PRRSV antibodies were detected by a competitive ELISA using a monoclonal antibody reactive for N-protein [30].

Serum antibody titres to PCV2 were determined weekly (from DAI −2 to date of euthanasia), whereas antibodies against PPV, ADV and PRRSV were determined at DAI −2 and at the date of euthanasia.

### Real–time polymerase chain reaction

Viral DNA obtained from swab, sera, seminal plasma and tissues was isolated using Trizol Reagent (Invitrogen, Carlsbad, CA, USA) according to the manufacturer’s instructions. The blood samples underwent RT-PCR for PCV2 following the protocol described by Olvera and others [31]. In addition, PRRSV, PPV and ADV genomes were tested for each blood sample by methods described by Bonilauri and others [32], Katz and Petersen [33], and Kim and others [34], respectively. No PPV, PRRSV and ADV genome were proved in swab and blood samples during the trial.

### PCV2-PCR for DNA sequencing

Total DNA was extracted from both the samples and viral stocks using a QiaAmp DNA Mini-Kit (Qiagen, Hiden, Germany) according to the manufacturer’s instructions. Amplification was carried out following the method proposed by Ouardani and others [35], incorporating forward and reverse primers ORF.PCV2.S4 and ORF.PCV2.AS4, respectively. The oligonucleotide set was designed to permit the amplification of a 493 bp long DNA fragment on the ORF-2 of PCV2 only. To confirm the same PCV2 strain used for infection, an additional PCV2a/PCV2b PCR was run on positive samples according to [25]. The PCR products were in both cases electrophoresed on a 2% agarose gel and visualized under ultraviolet light after staining with GelRed (Biotium, Hayward, CA, USA).

To confirm that the PCV2 detected in samples of infected animals was the strain used for infection, DNA sequencing was performed both on the PCV2 strain used for infection (positive control) and on the PCV2 DNA amplified by RT-PCR from positive cases. Prior to submission to the automated reaction to Eurofins MWG Operon, amplified products were cleaned up using a NucleoSpin Extract II kit (Macherey-Nagel, Düren, Germany). The sequences were analysed directly on the amplicons that were first aligned and then compared with known sequences present from NCBI GenBank (http://www.ncbi.nlm.nih.gov) [36].

### *Post mortem* sampling

Gross lesions were evaluated in the gilts and the following tissues were collected: tonsil, superficial inguinal, mesometrial and tracheo-bronchial lymph nodes, ileum, spleen, liver, kidney, ovary, salpinx, uterus, cervix, vagina, lung, and heart. All foetuses were numbered. From each foetus, amniotic fluid, allantochorion and adjacent endometrium, liver, spleen, and heart were collected. Aliquots of the sampled tissues were used for RT-PCR, histology and immunohistochemistry. During these procedures, sterilized instruments were changed between litters and each foetus to avoid cross-contaminations of samples.

### Histopathology and immunohistochemistry

Tissue samples were fixed in 10% buffered formalin, paraffin-embedded, and routinely stained with haematoxylin and eosin (HE). Immunohistochemistry (IHC) was performed using a PCV2 monoclonal antibody (PCV2 Mab F217) as previously described by Sarli and others [37], but using a streptavidin-biotin-peroxidase polymeric complex (SuperPicture kit peroxidise, Zymed® Lab, San Francisco, USA) to increase the test’s sensitivity.

## Results

**Figure 1:**
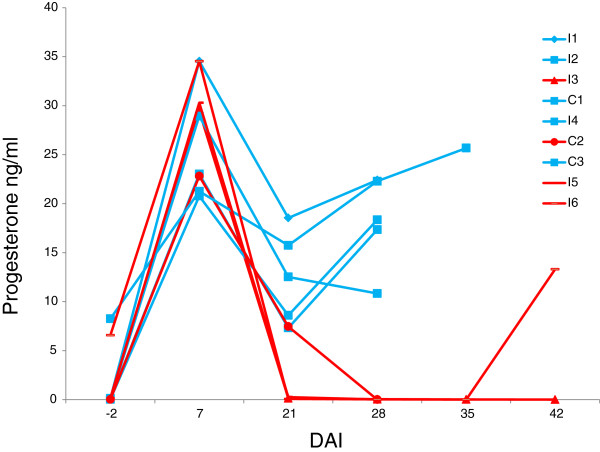
**Progesterone (P4) serum levels in individual gilts.** Levels of P4 in non-pregnant gilts are shown in red while blue represents pregnant gilts.

Sperm quality was not affected by the treatment with viral suspension. Overall motility and viability were both very high in control and viral exposed spermatozoa (respectively: 80.0% and 79.2% in control semen; 80.0% and 69.9% in exposed semen). In all the animals except C3 and I6, the P4 titre at DAI −2 was undetectable, but 40 h after Corulon® administration, only C3, which showed oestrus signs when synchronization started, did not display an evident cycle. Visible oestrus signs after AI were observed only in C3 DAI +15, when the animal was inseminated again. In this subject the day of the second insemination was considered DAI 0. At DAI +23, C2 aborted and three entire embryos were found on the box floor. At DAI +29, ultrasound proved pregnancies in five gilts: I1, I2, C1, I4, and C3. The three non-pregnant gilts (I3, I5, and I6) had not shown oestrus signs since DAI +21 but abortion had not been observed. As shown in Figure 1, gilts I3 and I5 did not show any variation in P4 titre up to DAI +42 and +28, respectively (oestrus was not observable before these time points) whereas P4 increased in gilt I6 after DAI +35 proving a previous silent oestrus ranging from DAI +35 to +42.

### *In vivo* results

At DAI −2, none of the gilts had viraemia for any of the tested viruses. Eight out of nine animals showed a higher anti-PCV2 antibody level at DAI −2 (range: 1/100 to 1/10000), only one gilt (I1) had a lower titre (1/100) (Table 1). Viraemia was assessed in four out of the six infected animals (I1, I3, I4, I5) in a +7 to + 35 DAI time range (Table 1). Gilt I1, with a low PCV2 antibody titre, had viraemia on DAI +21 and +35. For all PCV2 DNA positive sera, the viral load ranged from 1.10 × 10^3^ to 1.56 × 10^4^ PCV2 genome copies/ml. During the study only one faecal-positive swab (2.93 × 10^3^ PCV2 genome copies/ml) was found and obtained from gilt I1 on DAI +35. Analysis of the sequences detected in the infected group confirmed the identity of the amplified region of PCV2b used in the experimental infection and the virus subsequently isolated in the positive cases, showing 99% homology. Among the control subjects, only gilt C1 provided two viraemic blood samples on DAI +21 and +35. The sequencing of PCV2 DNA detected in gilt C1 demonstrated that it was a PCV2a strain.

**Table 1:** RT-PCR results of PCV2-infected foetuses and gilts

		**Number foetuses**	Serum titre against PCV2*	Viremia DAI**	**Tonsil**	**Left mesometrial lymph nodes**	**Right mesometrial lymph nodes**	**Tracheo- bronchial lymph node**	**Cervix**	**Foetuses (pos/tot.)**	**Amniotic fluid (pos/tot.)**	**Foetus and corresponding endometrium (left)**	**Foetus and corresponding endometrium (right)**
**Infected pregnant gilts**	**I1**	16	1/10^2^	21 and 35	-	+	+	+	-	10^§^/16	-	8***	1
	**I2**	11	1/10^3^	-	-	-	-	-	-	-	1/11	-	-
	**I4**	7	1/10^3^	21	-	-	-	-	-	3/7	-	-	-
**Infected non- pregnant gilts**	**I3**	0	1/10^4^	7	+	-	+	n.a.	-	-	/	-	-
	**I5**	0	1/10^4^	28	-	-	-	-	+	-	/	-	-
	**I6**	0	1/10^3^	-	-	-	-	n.a.	-	-	/	+	-
**Control gilts**	**C1**	11	1/10^3^	21 and 35^§§^	-	-	-	-	-	-	-	-	-
	**C2**	0	1/10^4^	-	-	-	-	-	-	-	/	-	-
	**C3**	8	1/10^4^	-	-	-	-	-	-	-	-	-	-

**Notes**:

“+” PCR-positive sample,

“-“ PCR-negative sample.

“n.a.” sample not-available.

* serum titres at the beginning of the experimental study.

** Days post artificial insemination/s of sampling when viraemia was recorded.

*** out of the 16 uterine tract samples, each corresponding to a foetus, 8 were PCV2 positive by PCR, but only in 7 cases there was concordance of the positive PCR result in both foetus and corresponding endometrium.

^§^ a foetus and its placenta were IHC positive, as well.

^§§^ both of them are PCV2a, strain not-used for the infection.

**Figure 2:**
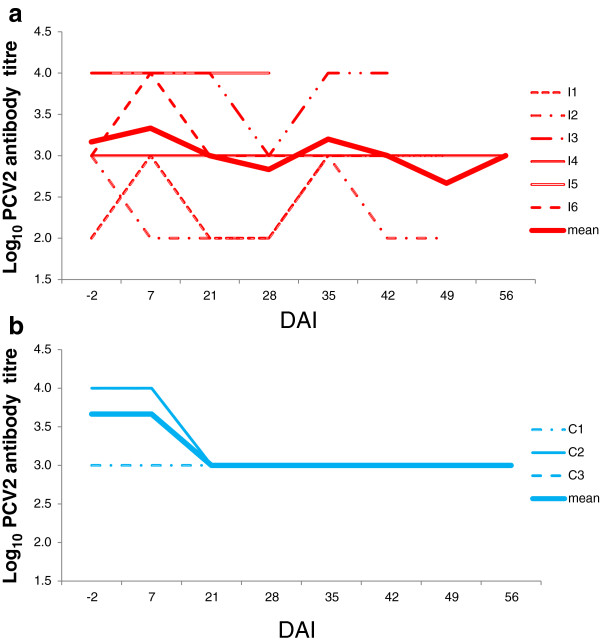
**a: Mean and individual anti-PCV2 antibody titres in inoculated gilts (IG). b:** Mean and individual anti-PCV2 antibody titres in control gilts (CG).

Anti-PCV2 antibody titre declined after DAI +7 followed by a plateau in the controls. The anti-PCV2 antibody titre showed non-synchronous seroconversion in four (I1, I2, I3, I6) out of the six infected animals resulting in a slight dynamic variation of antibody at the group level (Figure 2). Gilt I1 with the lowest anti-PCV2 titre (1/100) at the beginning of the experiment and another I2 that reached similar low values during the experiment, showed clear seroconversion over time and they also had PCV2 positive foetuses.

Serum antibody titres to PPV, PRRSV and ADV decreased between DAI −2 and at the last DAI (data not shown). These results suggest that no accidental infection with PPV, PRRSV and ADV occurred during the trial.

### Necropsy and *post mortem* results

The only lesions recorded included mild to moderate chronic fibrous pleuritis in four animals (I2, C1, C2, I5) and pericarditis in three gilts (I2 and C2 mild chronic fibrous and C3 slight serous pericarditis, respectively). One animal showed bilateral cranioventral bronchopneumonia (I4). One case with mild serofibrinous peritonitis (I3) and two with moderate multifocal interstitial hepatitis (white spotted liver) were observed (I5, C3). Three subjects displayed multifocal erosions in the stomach. The only uterine changes observed were mucosal congestion and oedema (gilts C2 and I6).

Gilts I1, I2, I4, C1, and C3 had 16, 11, 7, 11 and eight fetuses, respectively, with length and weight within normal range [38]. All foetuses appeared to have been alive when the gilts were euthanized and gross lesions were not observed.

Several tissues from infected animals were PCV2-positive (Table 1) with a viral load ranging from 1.01 × 10^3^ to 5.40 × 10^6^ PCV2 genome copies/ml. Placenta and foetus no. 16 of gilt I1 displayed the highest value of PCV2 genome copies/ml (4.01 × 10^8^). In the same gilt, the highest concomitance of PCV2 in both foetus and the corresponding uterine tract (8 out of 10) was recorded (Table 1). No gross or histopathological changes were identified and PCV2 was not detected by RT-PCR in the three aborted foetuses of the control gilt C2. Sequence analysis of the amplified region of PCV2 used for the insemination was the same as that isolated from tissues of infected animals.

**Figure 3:**
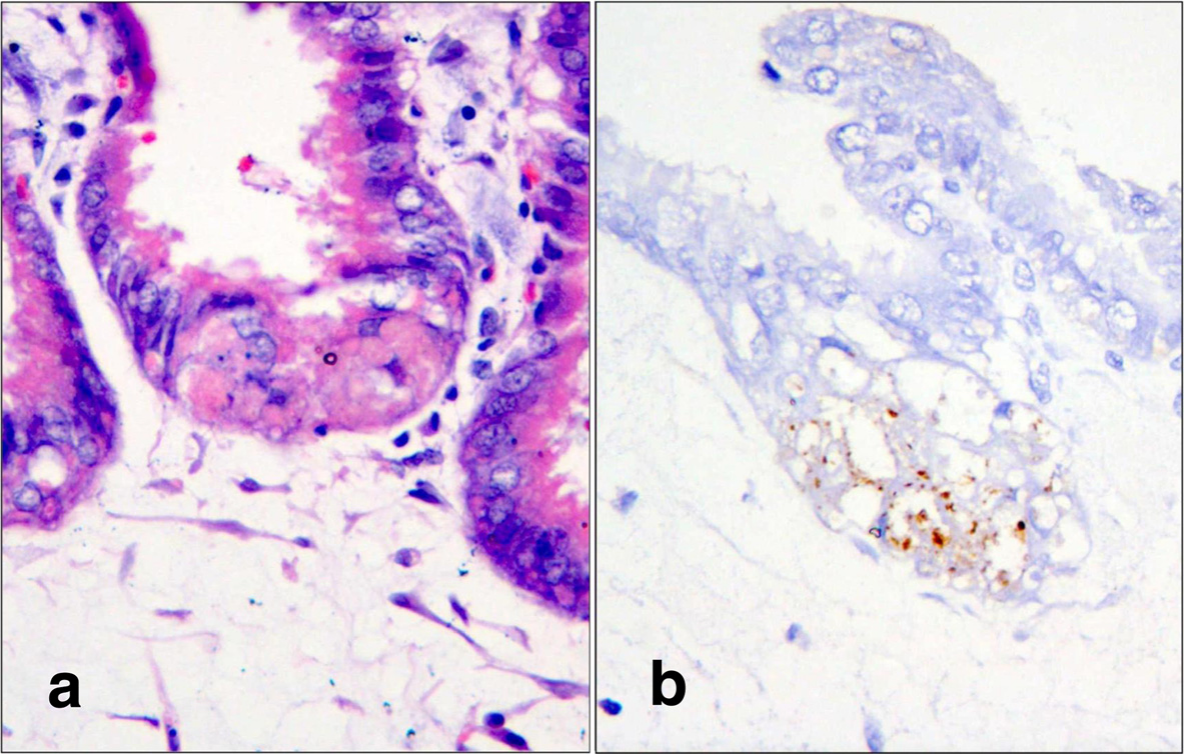
**Histological and immunohistochemical changes in the placenta of a PCV2b-infected porcine foetus.a:** Coagulation necrosis of trophoblasts. Haematoxylin and eosin, Obj. 40x. **b:** Immunohistochemical staining of PCV2b antigen in trophoblasts. Diaminobenzidine chromogen and haematoxylin counterstain, Obj. 40x. Photographs represent serial sections of a lesion with intralesional immunopositivity for PCV2 antigen.

Immunohistochemistry showed a weak cytoplasmic positivity for PCV2 in only one placenta positive for PCV2 DNA with the highest viral load (>10^8^/ml) with focal necrosis of the chorionic epithelium (Figure 3). The same foetus had an IHC-PCV2-positive reaction in the cytoplasm of hepatocytes after staining of liver sections.

## Discussion

Although the conditions created in this trial are experimental with a small number of animals, they reflect the situation recently described in a breeding sector of a piggery [39] in which 18.2% of tested semen were PCV2-positive. A field condition with intrauterine PCV2 inoculation, such as that occurring with contaminated semen [20], was investigated employing conventional gilts. The wide range in anti-PCV2 antibody titres of gilts in this study probably reflects the serological situation in a conventional herd.

The inoculation was successful: viraemia was recorded in four out of six inoculated gilts although weekly sampling is insufficient to identify viraemic phases in detail. More frequent sampling would probably have identified viraemia in more animals, e.g. in gilt I2 that had a PCV2 positive foetus and therefore had become infected. The anti-PCV2 antibody titre showed dynamic variations in infected subjects whereas a decline in anti-PCV2 antibody titre occurred after DAI +7 followed by a plateau in the controls. Although we cannot completely rule out that the virus shed by the viraemic gilts may have been transmitted to other gilts, the few viraemic events and the biosecurity measures adopted make this risk minimal. All control gilts became pregnant but gilt C2 aborted on DAI + 23, but without trace of PCV2b infection. Three out of six inoculated animals did not become pregnant but showed no signs of a subsequent oestrus. The plasma concentration of progesterone in gilt I6 at DAI −2 showed that the gilt had already ovulated, while gilts I3 and I5 had an optimal endocrinological status for fertilization, similar to that of the other subjects. The lack of pregnancy and the simultaneous infection (viraemia) in both subjects could involve PCV2 and its possible role in embryonic death.

Only the infected gilt with low PCV2 antibody titre at DAI −2 (I1) showed concomitant viraemia and faecal virus excretion. Presumably, the uterus is not a target organ and after transient replication in the uterus, PCV2 reaches the lymphoid organs and viraemia ensues. These viraemic episodes are the major source of foetal infection [40]. The spread of the virus to lymphoid tissues and the subsequent viraemia is favoured by low anti-PCV2 antibody titres. Because most of the foetal exposure to virus occurs after a maternal viraemic episode, antibody levels play a role in reducing the amount of virus that can cross the placenta [14,15]. Furthermore, other studies [40,41] imply that anti-PCV2 antibodies seem to be a partial safeguard against in utero PCV2 infection. The present investigation also suggests anti-PCV2 antibodies play a role in foetal protection even in the case of a primary intrauterine infection. Gilt I1, which showed the lowest anti-PCV2 titre at the beginning of the experiment and gilt I2 that reached the same antibody level at DAI +7, had PCV2b positive foetuses. It is known that intra-uterine exposure of foetuses to PCV2 does not produce infection in all of them and when spread occurs adjacent foetuses are usually affected [10,13,22,23]. The longer the infection lasts, the higher the probability of virus transmission [21]. The present results suggest that the proportion of foetuses infected by vertical transmission depends on the maternal antibody titre. The introduction of virus by semen is not the prime cause of viral exposure of the conceptus as long as the ZP protects it from PCV2 infection [16], but it is important for the development of a viraemia under which the foetuses become infected. This was confirmed by Madson and others [15] who showed that infection by artificial insemination can produce PCV2-seropositive piglets. The low antibody level can also influence the number of infected foetuses as indicated by the concomitant presence of PCV2 in both the foetuses and the corresponding uterine tract (Table 1).

The results demonstrate that PCV2 not only replicates in the foetus itself [7,8,13,14], but also in the chorionic epithelium where it probably causes focal necrosis. If sufficiently severe, placental lesions can cause foetal death, and replication in the placenta may expose the foetus to increased amounts of virus. These conditions may lead to stillbirth, mummification or abortion as observed under field conditions.

## Conclusions

This study supports the hypotheses that PCV2-seropositive gilts can be infected with PCV2 after intrauterine exposure and that a low maternal antibody titre may increase the probability of foetal infection.

## Competing interests

FJ and TV, who are employed by Merial SAS-Lyon France, participated in the design of the study. GL is employed by Merial Italy and participated in blood and swabs collection. Merial employees were not involved in other types of data collection during *in vivo* and *post mortem* phases, sample analysis or data interpretation. The manuscript was written independently of Merial employees. None of the other authors of this manuscript have any conflict of interest to declare.

## Authors’ contributions

GS, GA, TV and FJ conceived the study, participated in its design and study coordination. GL, DF, CaB, and MLB performed all the activities of the *in vivo* study. FO, MD, PB, DL, and SP performed and supervised the interpretation of serology and PCR investigations. GG and MLB performed semen evaluation and progesterone analysis. FM, LF, BB, and CiB did the *post mortem* investigations, including histology and immunohistochemistry. FM performed the statistical analysis, while FM, GS, and SP drafted the manuscript. All authors read and approved the final manuscript.
